# Genetic Diversity and Population Structure of the Rare and Endangered Plant Species *Pulsatilla patens (L*.*) Mill* in East Central Europe

**DOI:** 10.1371/journal.pone.0151730

**Published:** 2016-03-22

**Authors:** Monika Szczecińska, Gabor Sramko, Katarzyna Wołosz, Jakub Sawicki

**Affiliations:** 1 Department of Botany and Nature Protection, University of Warmia and Mazury in Olsztyn, Olsztyn, Poland; 2 Department of Botany, University of Debrecen, Debrecen, Hungary; 3 MTA-ELTE-MTM Ecology Research Group, Budapest, Hungary; 4 Department of Biology and Ecology, University of Ostrava, Ostrava, Czech Republic; Technical University in Zvolen, SLOVAKIA

## Abstract

*Pulsatilla patens* s.s. is a one of the most endangered plant species in Europe. The present range of this species in Europe is highly fragmented and the size of the populations has been dramatically reduced in the past 50 years. The rapid disappearance of *P*. *patens* localities in Europe has prompted the European Commission to initiate active protection of this critically endangered species. The aim of this study was to estimate the degree and distribution of genetic diversity within European populations of this endangered species. We screened 29 populations of *P*. *patens* using a set of six microsatellite primers. The results of our study indicate that the analyzed populations are characterized by low levels of genetic diversity (*H*_*o*_ = 0.005) and very high levels of inbreeding (*F*_*IS*_ = 0.90). These results suggest that genetic erosion could be partially responsible for the lower fitness in smaller populations of this species. Private allelic richness was very low, being as low as 0.00 for most populations. Average genetic diversity over loci and mean number of alleles in *P*. *patens* populations were significantly correlated with population size, suggesting severe genetic drift. The results of AMOVA point to higher levels of variation within populations than between populations.The results of Structure and PCoA analyses suggest that the genetic structure of the studied *P*. *patens* populations fall into three clusters corresponding to geographical regions. The most isolated populations (mostly from Romania) formed a separate group with a homogeneous gene pool located at the southern, steppic part of the distribution range. Baltic, mostly Polish, populations fall into two genetic groups which were not fully compatible with their geographic distribution.Our results indicate the serious genetic depauperation of *P*. *patens* in the western part of its range, even hinting at an ongoing extinction vortex. Therefore, special conservation attention is required to maintain the populations of this highly endangered species of European Community interest.

## Introduction

In the past centuries, habitat modifications induced by climate change and human activity led to the extinction of many plant species or severely confined their geographic ranges [1, 2]. Many plants species that had a continuous distribution range in the early 20^th^ century have since greatly retreated and were subsequently designated as rare and threatened species. In an era of rapid habitat loss and climate change, one of the key goals in species preservation and conservation genetics is to deepen our understanding of environmental forces responsible for shaping genetic diversity [3].

Species protection programs targeting large areas should focus on the most valuable populations to deliver tangible effects. The initiated actions require a thorough understanding of species biology and ecology as well as knowledge of the levels and distribution of genetic diversity [4]. Genetic diversity is an important consideration in species conservation because it influences a population's ability to adopt to a changing environment [5–7]. Dwindling populations of rare and endangered plant species are often characterized by low levels of genetic diversity [8, 9]. The above can be attributed to the fact that genetic diversity within an endangered population is lost due to relatively faster genetic drift, which is exacerbated by limited gene flow. This can lead to inbreeding depression and higher homozygosity, which results in reduced adaptive potential [5, 10, 11]. Genetic diversity is often correlated with plant fitness, and more genetically diverse populations are also more fit [5, 9, 12].

Determining the populations of the highest conservation value is not that simple. Knowing the level of genetic diversity, the question arises; what genetic criteria should be used to define the priorities? Allelic richness is a straightforward measure of genetic diversity, that is commonly used in studies based on molecular markers that aim at selecting populations for conservation [13, 14, 15]. This is especially important from a long-term perspective, because the limit of selection response is mainly determined by the initial number of alleles regardless of the allelic frequencies [16, 17] and because it reflects better past fluctuations in population size. Numerous studies in the field of conservation genetics show that priority for conservation should be given to populations that retain locally common alleles, these alleles that occur in high frequency in a limited area and can indicate that presence of genotypes adapted to specific environments [18].

The distribution of genetic diversity also plays an important role in species conservation [19–22]. Under circumstances of limited resources and possibilities for protecting all natural populations, which is usually the case in the real world, conservation efforts have to focus on selected populations. Genetic diversity within and between populations has to be identified to select populations that are responsible for the majority of the existing variation. If genetic variation is found mainly within a population, then fewer populations are required to protect and maintain the overall variation in the geographic range of a given species. If genetic variation is maintained mainly between populations, then a higher number of populations which constitute evolutionary units should be prioritized for protection.

In rare and endangered species most of the most threatened populations are peripherals [23]. Nonetheless, it is often debated whether conservation emphasis should be placed on peripheral or central parts of the distribution range [22, 24, 25]. It can be assumed that, in accordance the central marginal model [24], with populations at the edge of the species distribution expected to have lower genetic diversity and higher genetic differentiation than central populations [26, 27, 28]. This will be further exacerbated if peripheral populations experience more rapid cycles of extinction, recolonization and associated founder events or severe populations bottlenecks than those in less extreme central environments. The resulting stochastic reduction of genetic diversity within populations at geographical range marginal may limit their evolutionary potential thus hindering adaptation to conditions beyond the range limit [29, 30, 31]. However, a review published in 2008 found that this expectation was supported in only about two-thirds of studies that had compared core and peripheral populations [32]. Therefore the effects of both fragmentation and situation (peripheral versus core) on genetic diversity of populations can be difficult to predict.

Understanding the patterns and processes associated with geographical variation across species ranges is also motivated by conservation concerns. Geographically peripheral populations are often rare representatives of relatively widespread species within political jurisdictions [33]. Whether these range edge populations merit to conservation effort have been widely debated [22, 24, 25]. If peripheral populations are genetically impoverished owing to chronic genetic drift and low gen flow, then perhaps they are of little significance in terms of future evolutionary potential. On the other hand peripheral populations maintain substantial genetic variation they may adaptively diverge from more central populations owing to different selective pressure and reduced gen flow [34]. This populations may play role in the maintenance and generation of biological diversity [23, 35]. Information about the levels and distribution of genetic variation is not available for many priority, rare and endangered species. This deficiency hinders the development of effective conservation plans and the selection of populations for management strategies that aim to protect and maintain populations or increase genetic diversity.

*Pulsatilla patens* (L.) Mill. (*Ranunculaceae*) is one of such species. It is regarded as a rare and endangered species across Europe [36, 37–46]. The western boundary of its geographic range intersects central Europe (Poland) where the highest number of localities have been noted [36, 47] in the area. Despite the above, the size of *P*. *patens* populations has been dramatically reduced in the past 50 years, and the present geographic range is highly fragmented [36–38, 48–53]. In most confirmed localities, population size is limited to several sterile specimens. Similar reports are known from Germany [48], the Czech Republic [37] and Hungary [38], all at the very edge of the distribution. The single remaining Hungarian population is at high risk of extinction, and an *ex situ* garden population had to be established to maintain the plants in the wild [39]. The rapid disappearance of *P*. *patens* localities and populations from the European continent has prompted the European Commission to initiate active measures aiming to protect this critically endangered species. *Pulsatilla patens* is protected under Annex I of the Bern Convention [40], and it is listed in Annex II and Annex IV of the Habitats Directive of the European Union [41]. It is difficult to identify what are the reasons for the disappearance of the populations of *P*. *patens*. There can be both natural and antropogenic reasons behind. It is believed that one of the reason for the disappearance of the populations of *Pulsatilla patens* is presumably to a great extent related to changes in land use, especially in forestry practices where efficient wildfire prevention and termination of cattle grazing in forests has lead to the formation of a continuous moss layer or a strongly grass-dominated vegetation, which severely hinders the regeneration of this species [53, 54] Several factors are assumed to negatively affect the condition of the populations, including grazing on flowers and fruit-bearing shoots by animals [53], reduced seed production due to locally decreased number of pollinating insects [55], and unfavorable weather conditions such as long and freezing cold winters [56].

Small populations can be expected to demonstrate signs of genetic depauperation due to genetic drift. In comparison with annual and self-pollinating plant species, *P*. *patens* should be able to maintain higher levels of genetic diversity within a population and lower levels of genetic diversity between populations due to its specific breeding system, which promotes cross-pollination via insect pollination, and a long life-cycle. The present study was undertaken to determine whether habitat fragmentation and decreasing population size have influenced the genetic structure and diversity of *P*. *patens*. An analysis of genetic diversity will expand our knowledge of the species' reproductive strategy and provide valuable information for protecting and managing *P*. *patens* populations.

The aim of this study was to evaluate the distribution of genetic diversity in east Central European populations of *Pulsatilla patens*, a critically endangered species in most European countries. Microsatellite markers were used to determine: (1) the distribution of genetic variation across regions, populations and individuals, (2) whether analyzed populations of *P*. *patens*, which are small and influenced by stochastic processes are characterized by lower levels of genetic diversity, and (3) *P*. *patens* populations with priority conservation value in central Europe.

## Materials and Methods

### Ethics statement

The Polish populations were collected by MS with the permission given by General Directorate of Environmental Protection in Poland and the directors of Biebrza, Białowieża and Wigry National Park. The remaining populations were collected by GS with the assistance of L. Bartha (Cluj-Napoca), B. Lesku (Debrecen), B.A. Lukács (Debrecen), M. Russin (Kiev), I. Sramkóné Gáspár (Debrecen), R. Šuvada (Rožnava), A Szabó (Cluj-Napoca), V. Virók (Aggtelek). These plants were mostly collected from protected areas, and the sampling of a 1–2 cm^2^ leaf-segment was done with the consent of the local nature conservation authority in the presence of a representative person.

### Species analyzed

From a taxonomic point of view, *Pulsatilla patens* (L.) Mill. (Ranunculaceae) is a polytypic species with several taxa classified under this name either at the rank of subspecies or as species of subsection *Patentes* Aichele and Schwechler [57]. *P*. *patens* sensu lato (s.l.–in a broad sense) is a lowland species with a circumpolar geographic range [49], and it is found from central Europe (Germany, Poland) in the boreal and steppic zones through whole Eurasia to the prairies of North America. The taxon occurring in Europe, *P*. *patens* sensu stricto (s.s.–in a strict sense), occupies the western part of the area, and extends from Romania on the south to S Finland on the north, and from Germany on the west to W Siberia on the east, where it is replaced further to the east by *P*. *flavescens* (Zucc.) Juz. and *P*. *multifida* (Pritzel) Juz. [50, 58].

The European species, *Pulsatilla patens* s. s. is an early flowering hemicryptophyte [54] which produces a weakly branched tap root with hairy ground shoots. It usually flowers from the turn of March and April to the beginning of May depending on the altitude. The plant is characterized by xenogamous protogynous flowers which are mostly pollinated by insects of the family Apidae, bees [59]. Despite the presence of xenogamous flowers, self-pollination may occur due to the overlap in stigmas being receptive and pollen-shedding of the same flower [11, 59]. Nevertheless, autogamy produces significantly reduced fruit- and seed-set in the species [60].

As can be implied from the long life-span of the species, the generation time and yearly flowering vigor and fruit-set depend strongly on environmental and climatic conditions [53, 54, 61]. Flowering generally begins after several years at the earliest; botanical garden observation shows this can be at the age of two or three years. Specimens in open places usually produce the most flowers [53], but such individuals quickly decay. Habitat characteristics, such as thick moss cover, large amount of accumulated litter affects negatively the flowering by both reducing the number of flowering individuals and flowers on a single plant [54]. Seed production is high-approximately 60% of flowers were found to set fruit in an Estonian study [54]. Seeds germinate effectively only in areas with open patches in the vegetation [53–54, 60–62]. Climatic factors–especially springtime precipitation, temperature and sunlight–can have a very strong temporary effect on flowering, fruiting and even production of vegetative organs; populations can show more vigor in favorable years, but they can rapidly return to previous condition in a successive season [53].

Based on vegetative and reproductive characteristics, Kalliovirta et al. [52] distinguished three types of life-span stages in *Pulsatilla patens* populations: i) *increasing*, where relatively many rosettes are found in the smallest size classes, the proportion of seedlings is high and the proportions of generative plants is *ca*. 10%; ii) *stable*, where the proportion of rosettes in various life-cycle stages remain almost stable; iii) *decreasing*, where vegetative adults account for the vast majority (96%) of all rosettes and seedlings are usually completely absent. Most of the analyzed populations on the northern part of the area ('Baltic') represented this third type.

In the western part of the European distribution, the highest number of localities have been reported in Poland, and only isolated localities of *P*. *patens* s.s. have been noted in Estonia, Latvia, Czech Republic, Slovakia, Hungary and Romania [42–45, 48, 61]. The species is more widespread in Russia, although the plant is sporadic there as well [51]. *P*. *patens* is protected in European countries where it is regarded a critically endangered species. The species is listed in Annex II and Annex IV of the Habitats Directive of the European Union [41]. The plant has a preference for dry, sun-exposed habitats. Large parts of its geographic range cover steppe and forest steppe, and in Europe, *P*. *patens* is found mostly in pine forests.

### Analyzed regions and sampling protocol

**Fig 1:**
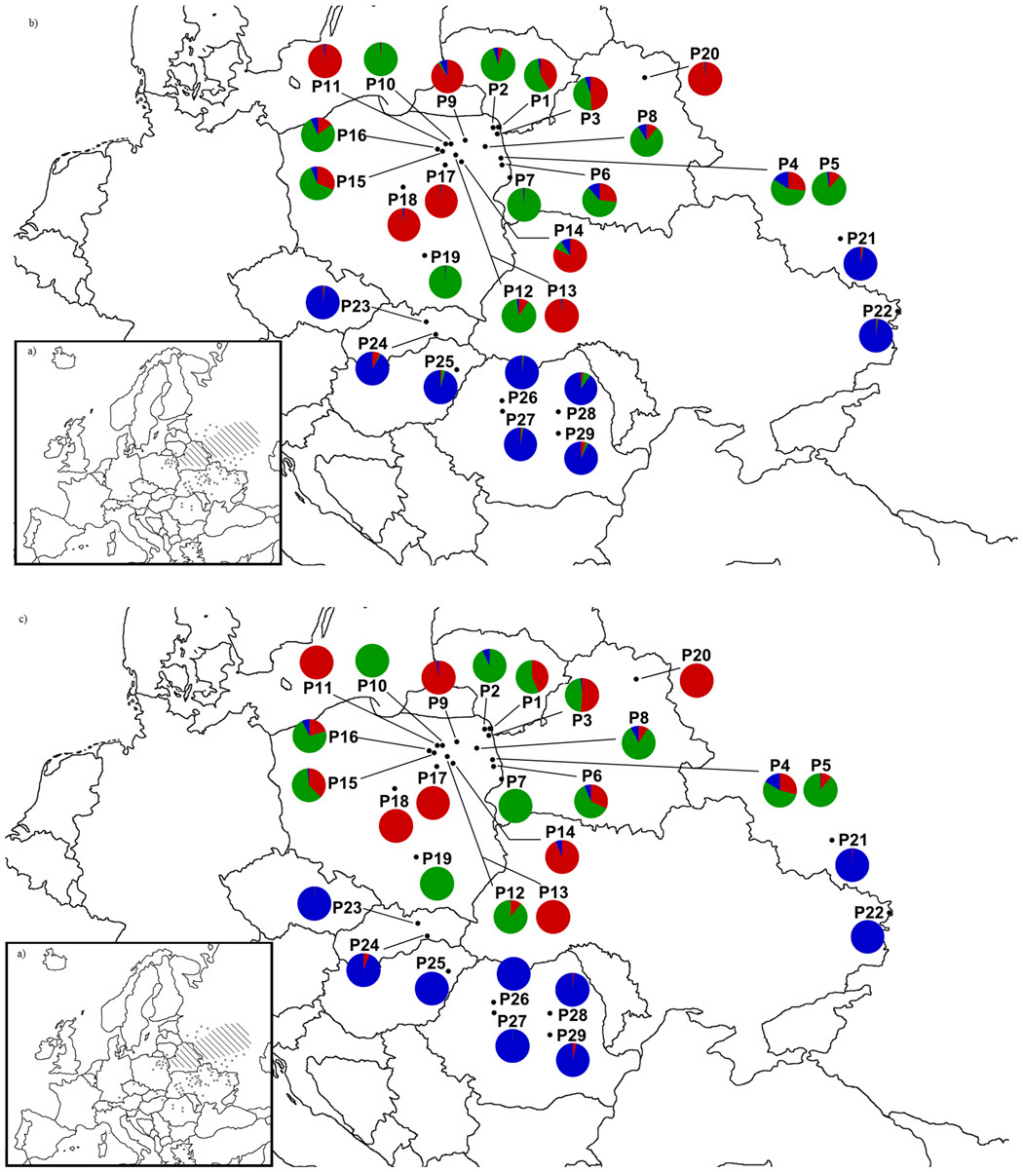
a) General distribution of *P*. *patens* in Europe (redrawn and slightly modified after Meusel et. al 1965). b) The location of 29 populations analyzed in this study. Each locations are represented by circles, and colors represent the genetic groups to which individuals within each site were assigned based on STRUCTURE results using K = 3 and a prior that takes sampling location into account–admixture analysis c) non admixture analysis.

For the present study, 29 populations of *P*. *patens* were sampled in central and eastern Europe: Poland, Belarus, Slovakia, Hungary, Romania, Ukraine, and Russia. (Fig 1a and 1b, Table 1). A population was defined as a group of plants separated from their nearest conspecifics by more than 1,000 m. The minimum distance between two locations was 3 km, and the maximum distance was 1652 km. Leaves from 595 individuals were sampled for the SSR analysis. A total of 5 to 62 samples were obtained from each population, and the number of samples was correlated with the size of populations. The minimum distance between collected samples within populations was 0.5 m.

**Table 1:** Geographical location of analyzed populations of *Pulsatilla patens* s.s.

Locality	ID	Country	Latitude N	Longitude E	Sample size in SSR analysis	Population size
Giby	P1	Poland	54°01’	23°12’	5	5
Wysoki Most	P2	Poland	54°02’	23°22’	14	15
Jazy	P3	Poland	53°53’	23°21’	23	24
Kopna Góra	P4	Poland	53°15’	23°30’	37	49
Łaźnie	P5	Poland	53°15’	23°48’	11	15
Żednia	P6	Poland	53°06’	23°32’	6	6
Białowieża	P7	Poland	52°47’	23°56’	5	5
Kopytkowo	P8	Poland	53°58’	23°56’	59	<800
Bemowo Piskie	P9	Poland	53°45’	21°59’	40	<300
Strzałowo	P10	Poland	53°38’	21°27	10	10
Spychowo	P11	Poland	53°37’	21°15’	12	15
Serafin	P12	Poland	53°20’	21°36’	19	19
Rudne	P13	Poland	53°23’	21°35’	16	27
Kolimagi	P14	Poland	53°21’	21°50’	62	<900
Zabiele	P15	Poland	53°27’	21°10’	36	36
Sasek Mały	P16	Poland	53°29’	20°53’	14	24
Parciaki	P17	Poland	53°7’	21°11’	9	9
Studzianka	P18	Poland	52°32’	19°24’	7	7
Bocheniec	P19	Poland	50°48’	20°18’	6	6
Witebsk	P20	Belarusia	55°14’	29°56’	28	56
Yamskaya Steppe Reserve	P21	Russia	51°11’	37°37’	23	>200 000
Stricovskaya Steppe Reserve	P22	Ukraine	49°17’	40°05’	13	>10 000
Primovce	P23	Slovakia	48°00’	20°23’	22	400
Hačava	P24	Slovakia	48°40’	20°49’	19	>10 000
Bátorliget^1^	P25	Hungary	47°46’	22°14’	16	20
Cluj-Napoca	P26	Romania	46°44’	23°32’	15	400
Rimetea	P27	Romania	46°27’	23°35’	22	1500
Frumoasa	P28	Romania	46°28’	25°54’	20	100
Sântionlunca	P29	Romania	45°49’	25°53’	20	>100 000

^1^ The samples analyzed are coming from the *ex situ* conservation garden of Hortobágy National Park

### SSR analysis

Total genomic DNA was extracted from 25 mg of silica-dried leaf material using the DNeasy^®^ Plant Mini Kit (Qiagen, Hilden, Germany). For populations P21–29, DNA was extracted following a modified CTAB-protocol detailed elsewhere [63]. Six microsatellite loci were used to analyze the genetic structure of the sampled populations. Microsatellite loci and PCR conditions used to generate data for the current study were described by Szczecińska et al. [64]. Electrophoresis was carried out using the QIAxel High Resolution Kit (Qiagen) with alignment marker 15–500bp and DNA size marker pUC18/Hae III for microsatellites. Automatic sizing of the amplified fragments was performed using a PC running BioCalculator software according to the manufacturer’s instructions (Qiagen, Hilden, Germany).

### Statistical analysis

Micro-Checker v.2.2.3 was used to test the genotyping of microsatellite data, identify genotyping and typographic errors and null alleles [65] using the default settings. For each population/locus we calculated frequency of null alleles using Brookfield’s estimator 2 [66]. Since undetected null alleles result in a reduced frequency of heterozygotes in the population, their presence would affect the output of tests for Hardy–Weinberg equilibrium (HWE). All estimates of population differentiation were carried out using both the real data and the corrected data set and the values. The results presented here were calculated using the corrected data set.

### Genetic diversity

The following genetic diversity parameters were calculated to determine the level of genetic variation within a population: mean number of alleles per locus (*A*), number of unique alleles, effective number of alleles (*Ne*), average observed heterozygosity (*Ho*), average expected heterozygosity (*He*), sample-size weighed expected heterozygosity (*UHe*), fixation index (*F*_*IS*_). All calculations were performed in GenAIEx v.6.5 [67, 68].

We calculated the presence/ absence of locally common alleles [14] in each population. The alleles have more than 5% frequency in each population and the same time occur in less than 25% of all populations examined. The average of locally common alleles was calculated using GenAIEx v.6.5 [67, 68].

Allelic richness (*Rs*) and private allelic richness (*pRs*) within each population were computed by the rarefaction method implemented in HP-Rare v.1.1 [69]. This approach relies on the frequencies of alleles at a locus to estimate the expected number of alleles and/or private alleles in a subsample of *N* individuals selected randomly from a sample of *N* individuals in each population.

The Hardy-Weinberg equilibrium (HWE) and linkage disequilibrium between loci were tested in FSTAT v.2.9.3 [70]. Significance levels were adjusted using Bonferroni correction for multiple comparisons. The correlation between genetic diversity indices and sample size were tested using Pearson’s correlation coefficient. The calculations were carried out in Statistica v. 19 [71]. The extent of population subdivision was evaluated by calculating Wright’s *F*_*ST*_ [72] and related *R*_*ST*_ [73]. Null allele-corrected pairwise F_*ST*_ estimates were calculated for all populations by applying the ENA correction in the FreeNA package [74, 75]. Uncorrected FST values were estimated following [74], whereas corrected FST estimates were made when null allele were predicted following the expectation maximization (EM) algorithm [76].

A genetic distance matrix of pairwise *F*_*ST*_ values was also used to perform a hierarchical analysis of molecular variance (AMOVA) [77] in Arlequin v. 3.11 [78]. Significance levels were determined using 1000 permutations. AMOVA was used to estimate and partition the total variances at three hierarchy levels: within populations, between populations and between groups of populations (geographic groups of populations based on two main regions identified by Bayesian methods at k = 3). The correlations between genetic and geographic distance (isolation by distance) [79, 80] were estimated for all populations by correlating *F*_*ST*_/(1-*F*_*ST*_) with geographic distance (km) in a Mantel test with 9999 permutations as implemented in GenAIEx. Bottleneck events were tested using two methods described by Cornuet and Luikart [81] and Garza and Williamson [82]. Infinite allele and stepwise mutation models were used, and significance was tested with the Wilcoxon signed-rank test in the program Bottleneck v. 1.2.02 [81, 83]. Principal coordinate analysis (PCoA) based on the pairwise *F*_*ST*_ distance matrix was carried out in GenAIEx. The Bayesian algorithm implemented in Structure v 2.3.3 [84] was used to analyze the genetic structure of the population. The percentage membership of each individual in every cluster was determined by the value of Q, and each individual was assigned to a specific cluster based on an arbitrary threshold of Q>0.70. We used two models; admixture (dependent allele frequencies) and non admixture (independent allele frequencies). The second model (non admixture) was used due the high inbreeding coefficient and the results showing that most of populations clearly deviate from being in HW-equilibrium. During the admixture analysis, K (unknown) group of populations are tested within a dataset, and each individual is assigned to one or more groups/clusters if the individual is admixed. Markov Chain Monte Carlo (MCMC) simulation values were set for a burn-in period of 100,000 iterations and run length of 1,000,000 iterations in an admixture model with correlated allele frequencies within populations. The batch run function was performed for a total of 140 runs (20 runs each for 1–32 clusters, i.e. K = 1–32) to quantify the variation in the likelihood of each K. The rate of change in log likelihood (delta K) between successive values of K was evaluated by the method proposed by Evanno et al. [85] to correctly estimate the number of clusters.

Likelihood and delta K values were visualized in Structure Harvester software [86]. Twenty simulation runs with the highest modal value of delta K were aligned in Clumpp 1.1 cluster matching and permutation software [87] and shown in a bar graph in Distruct 1.1 [88].

The number of groups were chosen after the Structure output files were analyzed in R software version 3.2.2 [89]. Similarity among results of different runs for the same K was calculated according to Nordborg et. al. [90] using Structure-sum 2009 [91].

## Results

### Genetic diversity

A total of 66 alleles were identified across all loci in 595 individuals. The presence of null alleles were observed in most loci analyzed. The frequency of null alleles ranged from 0,1671 in locus *Pul*04 to 0.3598 in locus *Pul*01 (S3 Table)

The highest number of alleles (15) was amplified by primers *Pul*01 and *Pul*04, whereas *Pul*10 produced the lowest value. The genetic diversity indices for six microsatellite loci calculated for each population are given in Table 2. The overall diversity in all populations analyzed was moderate; the average number of alleles per locus (*N*_*a*_) was 4.1±1.4 (mean±sd), the observed heterozygosity (*H*_*o*_) was 0.05±0.04, whereas expected heterozygosity was 0.56 ±0.13. Similarly, the measure of allelic richness (*R*_*s*_) also indicated genetically poor populations (3.25±0.67). In connection, the inbreeding coefficient (*F*_*IS*_) was considerably high, 0.92±0.06.

**Table 2:** Genetic diversity parameters for the 29 population of *Pulsatilla patens* s.s. studied divided into three genetic groups according to the Structure analysis: *N*–sample size; *N*_*a*_–number of alleles; *N*_*e*_ –effective number of alleles; *R*_*s*_ –allelic richness; *pR*_*s*_* –*private allelic richness; *H*_*o*_–observed heterozygosity; *H*_*e*_ –expected heterozygosity; *uH*_*e*_ –unbiased expected heterozygosity; *F*_*IS*_–inbreeding coefficient.

Name of population	Genetic cluster	Code	*N*	*N*_*a*_	*N*_*e*_	*H*_*o*_	*H*_*e*_	*uH*_*e*_	*F*_*IS*_	*R*_*s*_	*pR*_*s*_
Giby	Baltic 1	P1	5	2.50	2.11	0.000	0.493	0.55	1.00	2.5	0.00
Wysoki Most	Baltic 1	P2	14	4.66	3.21	0.033	0.658	0.681	0.95	3.87	0.00
Kopna Góra	Baltic 1	P4	37	6.00	4.59	0.000	0.748	0.758	1.00	4.42	0.00
Łaźnie	Baltic 1	P5	11	3.33	2.38	0.017	0.548	0.576	0.97	2.93	0.00
Żednia	Baltic 1	P6	6	2.83	2.34	0.000	0.497	0.543	1.00	2.81	0.00
Białowieża	Baltic 1	P7	5	1.50	1.24	0.033	0.137	0.152	0.70	1.50	0.00
Kopytkowo	Baltic 1	P8	59	8.16	4.15	0.076	0.718	0.724	0.91	4.42	0.07
Strzałowo	Baltic 1	P10	10	3.00	2.00	0.033	0.416	0.438	0.95	2.62	0.00
Serafin	Baltic 1	P12	19	4.00	2.79	0.009	0.624	0.641	0.98	3.27	0.00
Zabiele	Baltic 1	P15	36	6.16	4.56	0.102	0.740	0.751	0.88	4.43	0.00
Sasek Mały	Baltic 1	P16	14	4.33	2.90	0.119	0.594	0.616	0.85	3.49	0.00
Bocheniec	Baltic 1	P19	6	2.50	1.92	0.028	0.326	0.356	0.89	2.45	0.02
**MEAN**			**18,6**	**4.08**	**2.86**	**0.038**	**0.543**	**0.567**	**0.94**	**3.2**	**0.007**
Jazy	Baltic 2	P3	23	5.50	4.13	0.022	0.713	0.729	0.97	4.06	0.00
Bemowo-Piskie	Baltic 2	P9	40	4.66	2.87	0.050	0.619	0.627	0.93	3.35	0.00
Spychowo	Baltic 2	P11	12	3.66	2.72	0.000	0.560	0.585	1.00	3.24	0.00
Rudne	Baltic 2	P13	16	4.00	2.83	0.052	0.577	0.595	0.93	3.29	0.20
Kolimagi	Baltic 2	P14	62	7.00	4.55	0.062	0.747	0.753	0.93	4.38	0.08
Parciaki	Baltic 2	P17	9	3.66	2.65	0.074	0.488	0.516	0.91	3.21	0.09
Studzianka	Baltic 2	P18	7	3.50	2.82	0.048	0.611	0.658	0.93	3.37	0.01
Witebsk	Baltic 2	P20	28	5.00	3.63	0.071	0.692	0.705	0.91	3.74	0.02
**MEAN**			**24,27**	**4,62**	**3.27**	**0.047**	**0,626**	**0,646**	**0.94**	**3.58**	**0.05**
**MEAN (Baltic Group)**			**20.95**	**4.3**	**3.02**	**0.04**	**0.58**	**0.6**	**0.93**	**3.37**	**0.02**
Yamskaya Steppe Reserve	Southern	P21	23	3.83	2.68	0.080	0.530	0.542	0.90	2.91	0.00
Stricovskaya Steppe Reserve	Southern	P22	13	3.50	2.77	0.115	0.553	0.575	0.85	3.10	0.04
Primovce	Southern	P23	22	3.66	2.41	0.106	0.482	0.493	0.87	2.82	0.00
Hačava	Southern	P24	19	3.83	2.71	0.105	0.563	0.578	0.87	3.08	0.00
Bátorliget	Southern	P25	16	3.50	2.73	0.125	0.543	0.560	0.84	3.05	0.00
Cluj-Napoca	Southern	P26	15	3.16	2.36	0.044	0.548	0.568	0.93	2.81	0.09
Rimetea	Southern	P27	22	3.50	2.41	0.083	0.533	0.547	0.88	2.94	0.00
Frumoasa	Southern	P28	20	4.16	2.69	0.025	0.565	0.580	0.96	3.26	0.08
Sântionlunca	Southern	P29	20	3.83	2.61	0.075	0.524	0.537	0.90	2.98	0.00
**MEAN (Southern group)**			**18.89**	**3.66**	**2.6**	**0.08**	**0.54**	**0.55**	**0.89**	**2.99**	**0.02**
**OVERALL MEAN**			**20.31**	**4.1**	**2.89**	**0.05**	**0.56**	**0.58**	**0.92**	**3.25**	**0.02**

**Fig 2:**
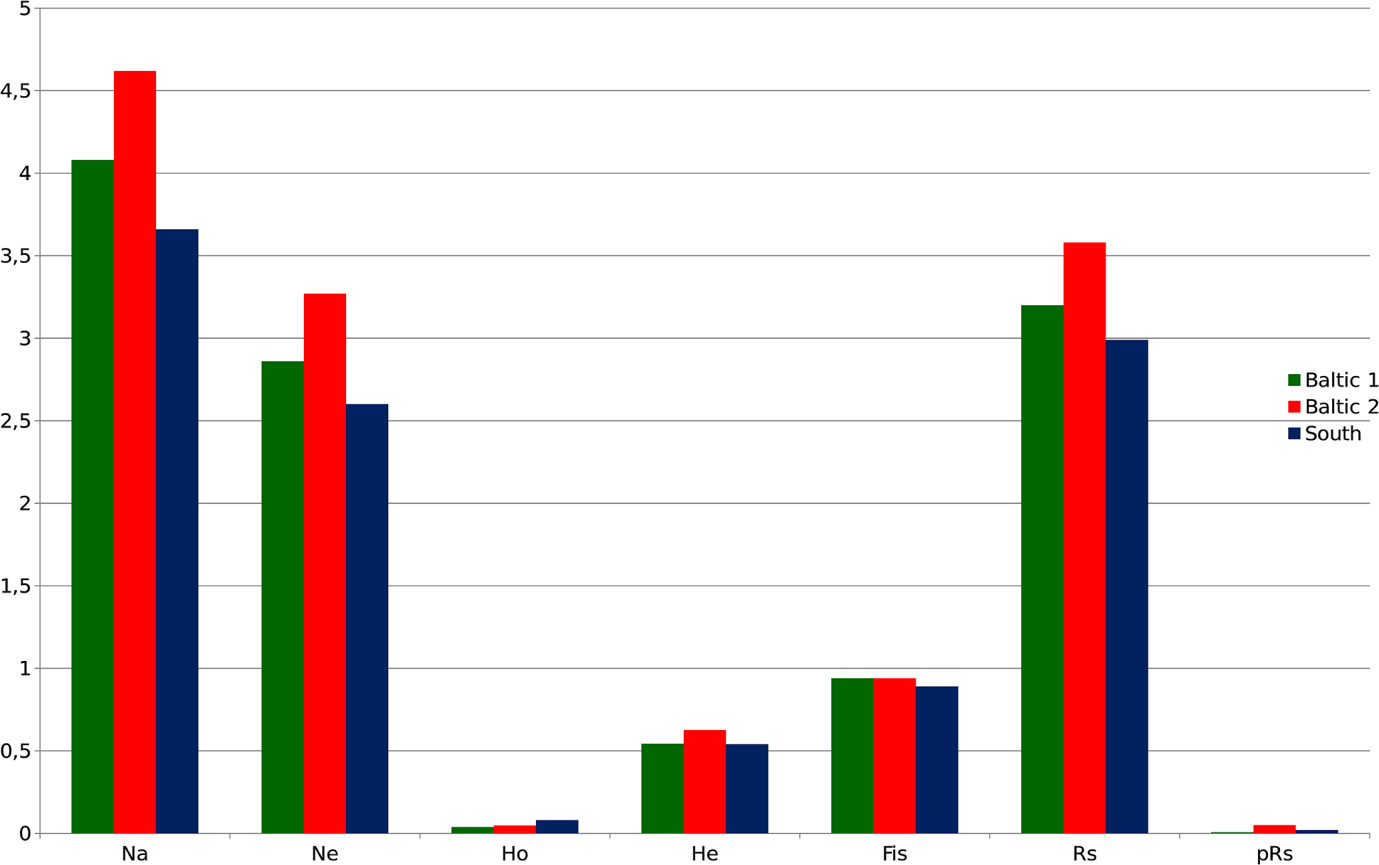
Mean genetic diversity parameters for three clusters identified by the STRUCTURE analysis.

**Fig 3:**
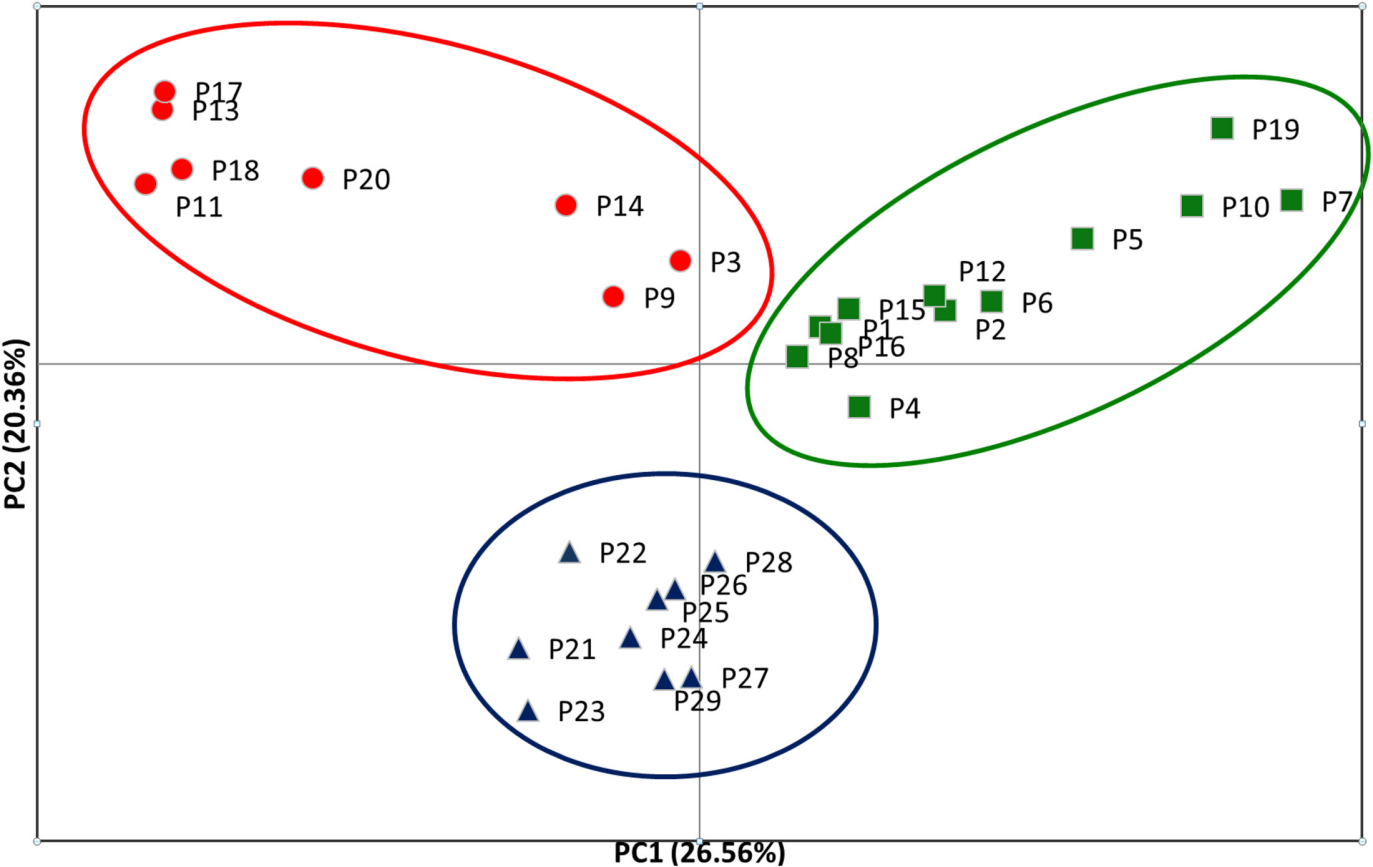
Principal coordinate analysis (PCoA) based on *F*_st_ genetic distances for 29 studied populations of *Pulsatilla patens* s.s. Encircled populations correspond to the three clusters identified by the STRUCTURE analysis. The symbols denote three genetic clusters.

The analysis of genetic diversity parameters in three genetic groups, which are distinguished in the Structure analysis (Table 2, Fig 2), pointed that the highest average number of alleles and mean expected heterozygoisity were found in the group‘Baltic 2’ (Table 2). This group of population also showed the highest allelic richness. The observed differences in the value of this parameter between three genetic group of the analyzed populations are not significant (Kolmogorov-Smirnoff test: p<0.01). The analysis of genetic distances revealed two main genetic groups (Fig 3): one consist of the Belorussian and all Polish ('Baltic') populations on the northern part of the area sampled, whereas the remaining samples from the southern part of the distribution area formed a more discrete, separate ('Southern') group. Interestingly, some differences in genetic diversity can be observed between the two groups of samples. Usually, higher absolute figures of genetic diversity and greater variation in data were obtained for the Balitc samples; all the minimal and maximal values of genetic diversity were found in this group except for *H*_*o*_, which was higher in the Southern group. The average number of alleles and expected heterozygosity values were actually higher in the Baltic populations, whereas the Southern populations were characterized by higher observed heterozygosity values and a smaller extent of inbreeding (*F*_*IS*_) (Table 2). Nevertheless, in statistical terms only observed heterozygosity was in fact significantly higher (Kolmogorov-Smirnoff test: p<0.01), all the other differences were either not or marginally significant (p<0.05).

The populations analyzed differed considerably in the evaluated genetic diversity parameters. SSR loci revealed moderate genetic diversity in the analyzed populations. The highest average number of alleles per locus was detected in population P8–Kopytkowo, NE Poland (8.1±1.108), whereas the lowest number of alleles was found in population P7–Białowieża, NE Poland (1.5±0.224). The studied populations were characterized by very low percentage of heterozygous individuals. The mean value of observed heterozygosity (*H*_*o*_) was only 0.04±0.03, and it ranged from 0.000 to 0.119 across the studied Baltic populations (Table 2). Observed heterozygosity differed considerably from expected heterozygosity which was much higher (*H*_*e*_ = 0.58±0.15) and ranged from 0.137 to 0.748 in the examined populations. A very small percentage of heterozygous individuals in the analyzed populations was confirmed by a high inbreeding coefficient (*F*_*IS*_ = 0.93±0.07). The highest values of *F*_*IS*_ were observed in four Polish populations (P1, P4, P6, and P11), whereas in the remaining populations F_IS_ statistics also indicates high values of inbreeding ranging from 0.70 (P7–Białowieża) to 0.988 (P12–Serafin, east-central Poland) (Table 1). Allelic richness values ranged from 1.50 (P7–Białowieża) to 4.42 (P4–Kopna Góra, P8 –Kopytkowo, both NE Poland) (Table 1). Private allelic richness, determined by the rarefaction method, was very low, being as low as 0.00 for most populations. Positive correlations were observed between *N*_*e*_ (r = 0.412, p = 0.024), *H*_*e*_ (r = 0.392, p = 0.032) and population size.

On the southern part of the distribution the genetic diversity values were more similar between the populations; the mean value of average number of alleles per locus (*N*_*e*_) was 3.66±0.29, observed heterozygosity (*H*_*o*_) was still low 0.08± 0.03, and ranged from 0.025 (P28–Frumoasa, E Transylvania) to 0.125 (P25–Bátorliget). As for the group mean expected heterozygosity (*H*_*e*_) we had 0.54±0.03, and the lowest value was exhibited by P23–Primovce, N Slovakia (*H*_*e*_ = 0.482), whereas the highest value was displayed by P28 –Frumoasa, (*H*_*o*_ = 0.565). As mentioned above, slightly less levels of inbreeding was characteristic for the Southern populations (*F*_*IS*_ = 0.89±0.04); the lowest value (*F*_*IS*_ = 0.84) was found in P25–Bátorliget, whereas the highest (*F*_*IS*_ = 0.96) in P28–Frumoasa.

### Genetic variation

The 371 pairwise genetic distances (pairwise F_*ST*_ values) between pairs of 29 analyzed populations were heterogeneous. It is ranged from 0.011 to 0.596 (mean F_*ST*_ = 0.219±0.041). The highest level of genetic differentiation was observed between populations P7 (Białowieża, E Poland) and P17 (Parciaki, central E Poland), whereas the lowest level of variation was observed in P2 (Rudne, central E Poland) and P15 (Studzianka, central Poland). Utilizing an ENA correction, the overall genetic differentiation and pairwise F*ST* were slightly lower (F*ST* = 0.189), indicating that null alleles only had a small effect on genetic differentiation (S4 Table).

The results of AMOVA point to higher levels of variation within populations (77%) than between populations (23%). Genetic variation between Polish populations of *P*. *patens* and populations sampled outside the core distribution range of the species was determined at 8%. It was estimated at 19% between populations within groups and 73% within populations. The STRUCTURE analysis of three genetic groups revealed somewhat higher variation between groups (11%) and lower variation within populations (74%) with *F*_*ST*_ = 0.25 (Table 3).

**Table 3:** Analysis of molecular variance (AMOVA) of *Pulsatilla patens* s.s. for 29 populations, two parts of geographical range and three genetic groups.

Source of variation	df	Sum of squares	Variance components	Percentage of variation	*F*_*ST*_
1. Total					
Among all population	28	714.130	25.50	23%	0.234***
Within population	1149	2222.058	1.934	77%	
2. Two parts of geographical range					
Among regions	1	114.784	114.784	8%	0.273***
Among populations within regions	27	599.346	22.198	19%	
Within populations	1149	2222.058	1.934	73%	
3. Three groups as in Structure					
Among groups	2	273.182	136.591	11%	0.259***
Among population within groups	26	439.235	16.894	15%	
Within populations	1147	2219.020	1.935	74%	

***p<0.001

**Fig 4:**
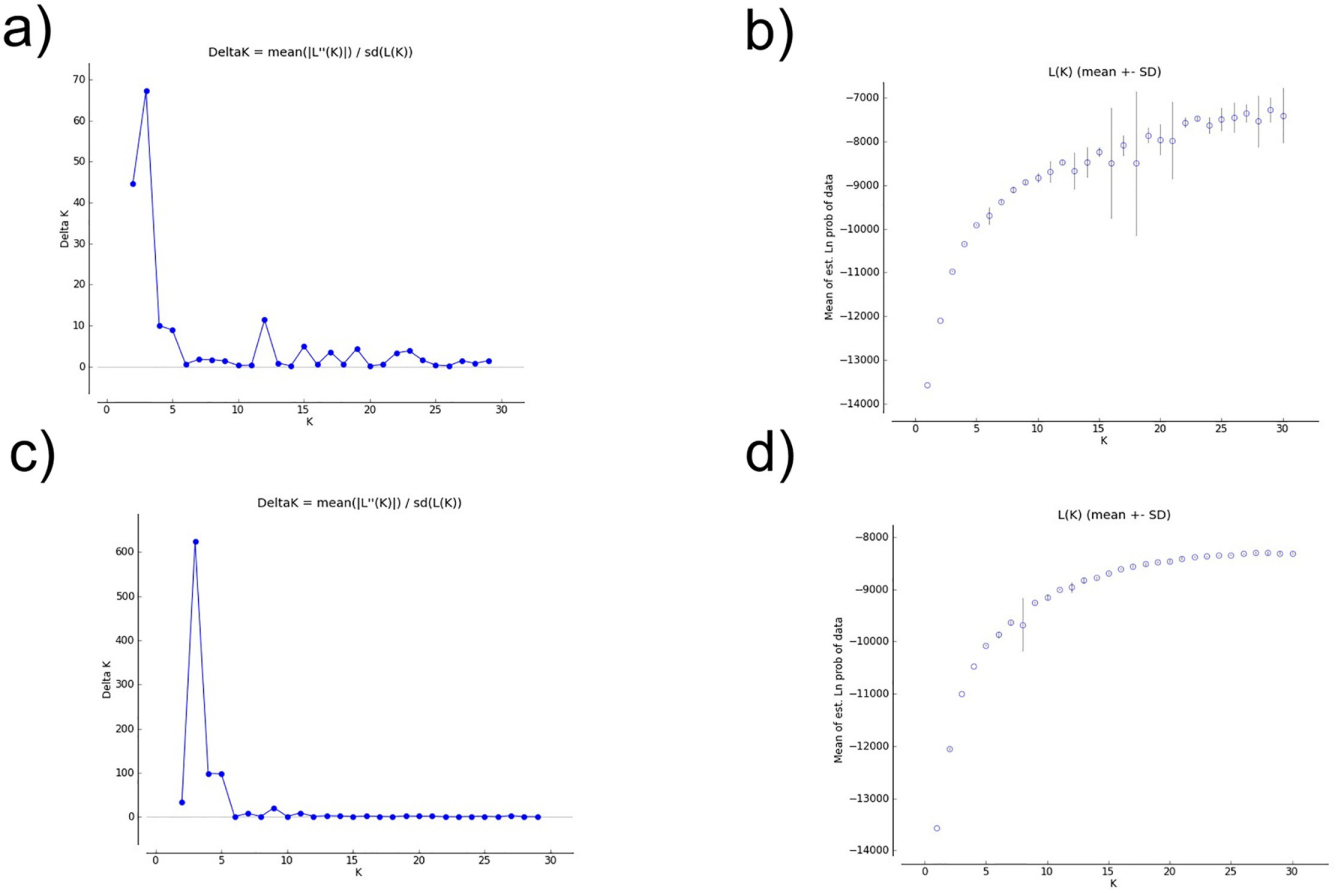
Results of the STRUCTURE analysis (10 runs each for K = 1–30) to infer population structure of studied *Pulsatilla patens* s.s. populations. a) values of Evanno’s admixture analysis; b) values of Evanno’s non admixture analysis; c) Posterior probabilities lnP(K)-admixture analysis; d) Posterior probabilities lnP(K)-non admixture analysis.

The population structure of *P*. *patens* inferred in Structure revealed that K = 3 was the most probable number of clusters for both the admixture and non-admixture models (Fig 4a, 4b, 4c and 4d), and similarity coefficients among replicate runs were highest at K = 3 (mean±SD, 0.999±0.002 -non admixture model, 0.993±0.002-admixture model).

**Fig 5:**
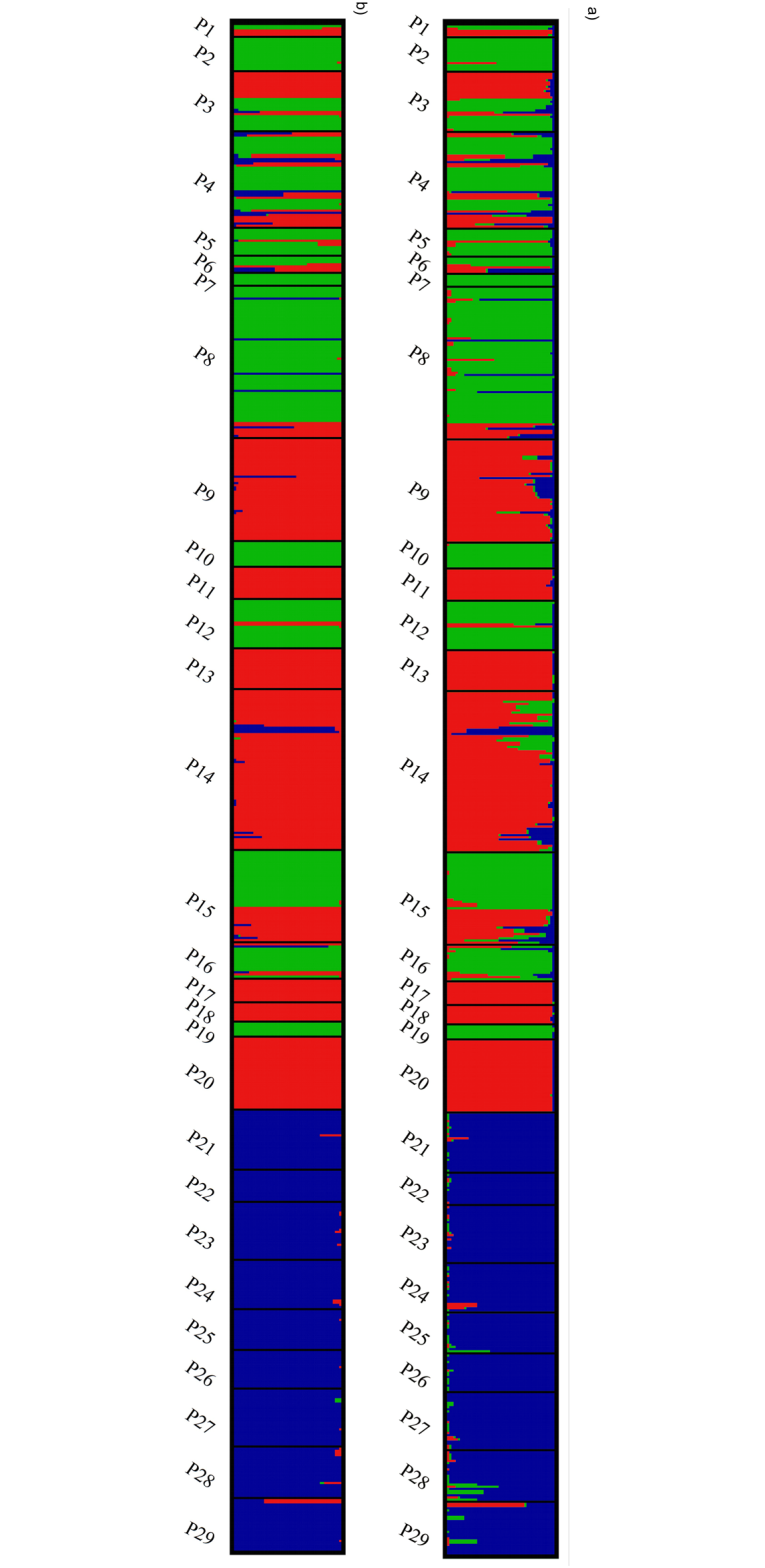
Population structure of 598 samples of *Pulsatilla patens* s.s. uncovered by STRUCTURE (K = 3); colors represent the groups identified; each individuals is represented by vertical line, each population is represented by one chart, showing the proportion of membership to the different clusters. a) admixture analysis; b) non admixture analysis.

The presence of three genetic groups was identified in *Pulsatilla patens* individuals, and in most cases, they were not influenced by geographic distribution (Fig 5a and 5b). For both of the models (admixture and non- admixture) we have not observed differences of individuals contribution to the distinguished clusters. The southern populations (P21–P29) had the highest Q value in the third cluster (Q_3_ = 0.90–0.981, 'Southern' cluster). The remaining two clusters were formed by the Baltic populations. The individuals from populations P1, P3, P4, P6 and P15 were characterized by admixed genotypes, and their Q values were indicative of membership in two clusters, cluster 1 (red) and cluster 2 (green), rather than a single cluster where Q>0.70. The majority of individuals from populations P9, P11, P13, P14, P17, P18 and P20 belonged to the first cluster, whereas most individuals from P2, P5, P7, P8, P10, P12 belonged to the second cluster (Fig 2).

Principal coordinate analysis (PCoA) based on Nei’s unbiased genetic distance (Fig 3) generally confirmed the separation of *Pulsatilla patens* populations as inferred by the Bayesian approach. Two main groups were found: one consisting of a very broad range of samples coming from the Baltic populations, whereas the other one was formed by a more discrete group of samples from the southern, steppic part of the distribution area. The first axis, which depicted 26.56% of the variance, separated the central Baltic populations of *Pulsatilla patens* into two distinctive groups. Three Polish populations (P3, P9, P14) occupied an intermediate position. The second axis, which accounted for 20.36% of molecular variance, clearly separated the southern populations of *P*. *patens* (Fig 3).

**Fig 6:**
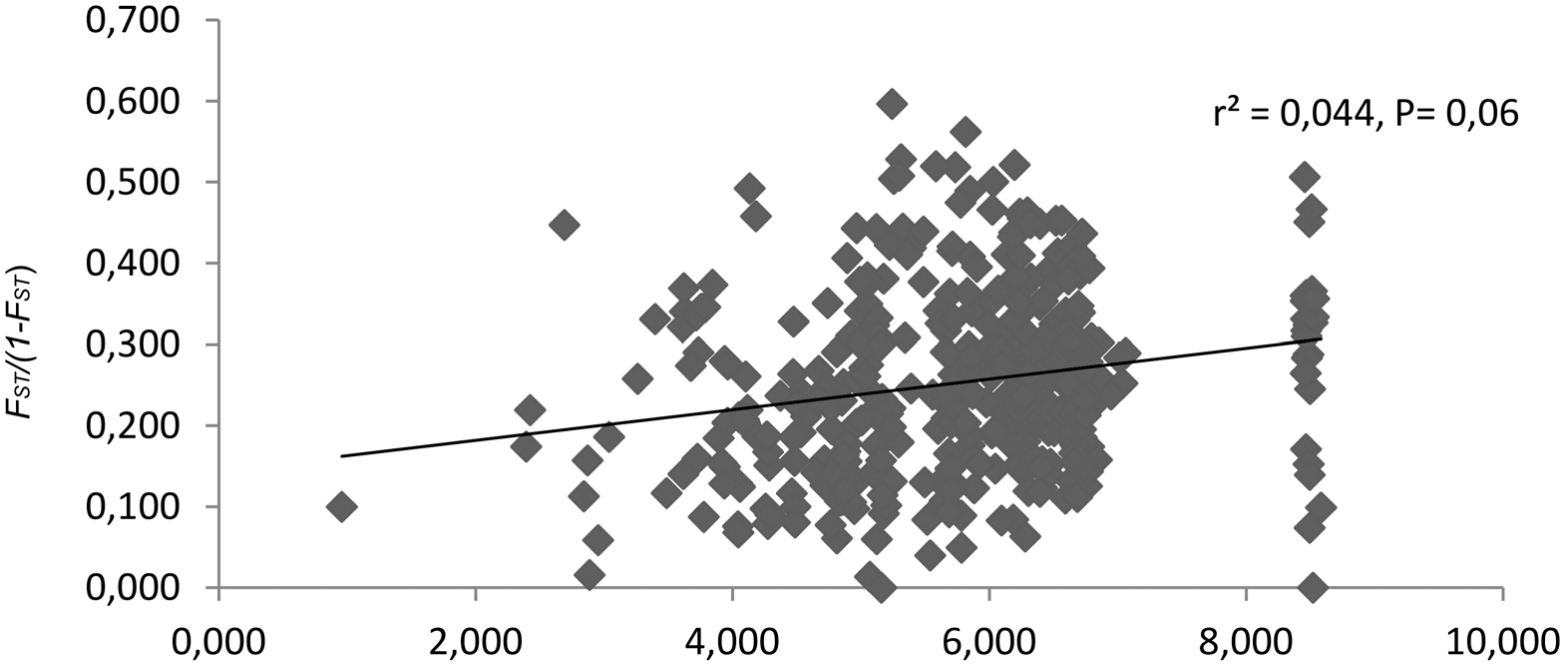
The relationschip between genetic distances (pairwise *F*_st_) and geographic distances for a 29 sampled populations of *Pulsatilla patens*. P. values were derived from Mantel tests.Note log scales for geographic distances.

We found no correlation between genetic distance and geographic distance as indicated by a Mantel-test (r = 0.004, P = 0.120) (Fig 6).

The differences between the three genetic groups could be attributed to the presence of unique alleles as well as differences in the frequency of the remaining alleles. The Southern group, which comprised isolated populations of *Pulsatilla patens* and was characterized by the most homogeneous gene pool, was devoid of 29 alleles that were found in populations from the entire geographic range sampled. These populations also had a different pool of locally common alleles which were characterized by low frequency within populations.

## Discussion

### Genetic diversity

The results of our study indicate that east central European populations of *Pulsatilla patens* are characterized by low levels of genetic diversity within populations. Very low levels of observed heterozygosity (*H*_*o*_ = 0.0547±0.04) in the analyzed populations point to high heterozygote deficiency and very high levels of inbreeding depression (*F*_*IS*_ = 0.917±0.06).

Numerous studies of rare and endangered plant species demonstrated that inbreeding can result from a reduction in population size which lowers genetic diversity and leads to inbreeding depression [92–95]. This is a highly probable scenario in the studied populations of *P*. *patens*, which is confirmed by recent reports of the species' continued disappearance [36–38, 49–53]. Similarly to other rare and endangered species [96, 97], inbreeding in *P*. *patens* populations could reduce plant fitness and promote a further drop in abundance.

In general, levels of genetic diversity and high fixation index in the present day populations of *Pulsatilla patens* may be more likely to reflect the populations accumulated history of the size fluctuation than be reflect to present population size. Despite the above, the size of *P*. *patens* populations has been dramatically reduced in the past 50 years, and the present geographic range is highly fragmented [36, 37, 38, 48–51]. In most confirmed localities, population size is limited to several vegetative specimens [36–38, 49–51, 53]. This populations could be exposed to greater effect of genetic drift, whose effect are observed in the low level of genetic diversity and high level of *F*_*IS*_.

One of the reasons of the low level of genetic differentiation and very high fixation index can be the location of most of the studied populations of *Pulsatilla patens* on the edge of the distribution range of this species and evolutionary processes that usually accompany peripheral populations [20, 24]. Fragmentation of the geographical range of *P*. *patens* has led to the isolation of the population and also prevented the efficient flow of genes. Although genetic differentiation as measured by *F*_*ST*_ between the studied population showed an average value (*F*_*ST*_ = 0.22), but noted that among the study populations were such for which this parameter has reached very high values.

High values of *F*_*IS*_ could be attributed to self-pollination in *P*. *patens* populations. Although the analyzed species is xenogamous, selfing can occur but probably this is not the real cause of the significantly lowered genetic diversity. Most of Europe is suffering from the general decline of pollinator insects (mostly bees), a phenomenon called pollination crises [98–100]. Although we do not have exact data from the regions where *P*. *patens* occurs and, probably more importantly, from the spring season when it flowers, one could expect an elevated rate of self-pollination due to the presumable lack of effective pollinators. Nevertheless, a recent study focusing on Hungarian orchids did not find evidence of pollination crisis in Hungary [101], but these results may not apply to Poland, and/or to springtime flowers like *P*. *patens*.

As for the two main groups of samples (i.e., the 'Baltic' and 'Southern' groups), there where slight and statistically non-significant differences in genetic diversity measures between the two groups. The only remarkable difference is the slightly lower levels of inbreeding in the Southern group, which might be connected to slightly higher number of pollinators at the lower latitude. In other respect, the lack of statistically significant differences between population genetic indicators hint at a similar rate of declining populations. Positive correlations were observed between most genetic diversity parameters and population size, what confirm earlier studies [8, 9, 102]. Small populations of *P*. *patens* comprising several individuals were characterized by the lowest levels of genetic diversity in comparison with the largest populations. Similar correlations were reported in populations of *P*. *vulgaris* [103]. Leimu et al. [9] gave two reasons for the positive correlation between population size and genetic diversity. Firstly, a positive correlation could imply the presence of an extinction vortex, where the drop in population size lowers genetic diversity and leads to inbreeding depression. This is a highly likely scenario in the analyzed populations of *P*. *patens* which was a common species in the past, but is now rapidly declining due to habitat changes partly caused by human activity. Habitat fragmentation, can be one of the reasons that lead to strong genetic drift. Besides anthropogenic factors, we have to consider the current climate change as a reason for decline of this otherwise cold-adapted species.

The second reason for the presence of a positive correlation between population size and genetic diversity is the fact that plant fitness differentiates populations based on variations in habitat quality [104, 105]. In the long-term perspective, a reduction in genetic variation could be expected to lower the adaptability of a population and increase the risk of its extinction under changed habitat conditions. The above observations are consistent with the results of our study where various genetic methods were deployed to determine the effect of population size on rare and endangered plant species [103–107]. Low levels of genetic diversity could reduce plant fitness and restrict a population's ability to respond to changing environmental conditions through adaptation and selection [108]. The vast majority of small populations were composed mainly of vegetative specimens, whereas large populations comprised individuals that produced flowers, fruit, seeds and seedlings. Nevertheless, the Southern populations sampled were seemingly in a good condition; most of the populations were characterized by large numbers of flowers, and large numbers of adults plants even in spatially restricted populations (e.g. P23–Primovce, N Slovakia; P26–Cluj-Napoca, central Transylvania).

### Differences within and between populations

Most genetic diversity (77%) was observed within populations, whereas 23% of genetic variation existed between the evaluated populations. The breeding system is one of the key factors determining the distribution of genetic variation in plant populations [109, 110]. In cross-pollinating plants, most of total genetic variability is distributed between individuals within a population, a smaller proportion of it is attributable to the variation between populations [111]. In AMOVA, the mean value of *F*_*ST*_ for the studied populations was 0.23, and it is comparable with the values reported for cross-pollinating and long-lived perennial species (*F*_*ST*_ = 0.22) [112].

Despite generally moderate differences between the evaluated populations, significant genetic variations were observed in selected population pairs. The highest differences (*F*_*ST*_ = 0.596) were noted between populations P7 (Białowieża) and P17 (Parciaki). Population P7 was generally characterized by significant genetic distinctness with mean *F*_*ST*_ of 0.488 (ENA correction) (S4 Table). The presence of significant genetic variations between *P*. *patens* populations separated by a distance of only several kilometers testifies low gene flow. The mean value of N_m_ was estimated to be 0.909, and it was lower than that reported in *P*. *vulagris* populations in central Germany (N_m_ = 1.22) [70]. Couvet [113] demonstrated that one migrant per generation may not be sufficient to guarantee long-term survival of small populations and that the number of migrants is determined by life history traits and population structure [114]. The majority of the examined *P*. *patens* populations were coming from the edge of the distribution, therefore, it can be assumed that gene flow between these populations is hampered. The observed differences were not correlated with geographic distance, however, and random differences in *F*_*ST*_ values could point to habitat fragmentation and the presence of barriers to gene flow between populations. In *P*. *patens*, gene flow could also be obstructed by the method of pollination and seed dispersal. *P*. *patens* is an insect pollinated species. Pollen-mediated gene flow is generally limited because most pollinators travel less than 20 km [70], and they tend to visit neighboring plants [115]. Plant populations may be geographically too isolated to be connected by pollen exchange. In *P*. *patens*, the manner of seed dispersal may also inhibit effective gene flow; in spite of having hairy achaenes adapted to wind-dispersal, most seeds land in the close vicinity of parent plants, and only dispersed on a longer distance by epizoochory [48]. A similar dependency was observed in *P*. *vulgaris* populations [103].

The considerable genetic differences between selected population pairs could also be attributed to the absence of generative reproduction and thus increased genetic drift [116, 117]. In the analyzed localities, long-term field observations ruled out generative reproduction in the majority of small *P*. *patens* populations due to the absence of flowering individuals or their inability to produce fruit. The above could have inhibited gene flow and exacerbated genetic differences. The observed genetic differences between selected population pairs could also be correlated with the small size of those populations. A limited number of individuals in a population (e. g., P6, P18, P7) inhibits effective gene flow. Genetic differences could also be attributed to genetic drift which is often noted in small populations [118–120]. Genetic drift could also be responsible for genetic differences observed in the studied populations.

The results of Structure and PCoA analyses suggest that the genetic structure of *P*. *patens* consists of three clusters comprising several populations each. The most isolated populations (P21–P29, the 'Southern' group) formed a separate group with a comparatively homogeneous gene pool. Baltic populations were more-or-less divided into two genetic groups which were not fully consistent with their geographic distribution. The easternmost populations of *P*. *patens* in large forests (Augustów Primeval Forest, Knyszyn Forest and Piska Forest, E Poland- P2, P4, P5, P6, P7, P10), harboring the largest populations of the studied species, formed the same group (cluster 2). The remaining populations, including numerous small populations, belonged to the most heterogeneous group 3. This group represents populations occupying the geographic margin of the species range where habitat fragmentation processes take place over much longer periods of time than in eastern populations.

The observed genetic differences between the three groups were relatively small, but statistically significant. The absence of differences or minor differences between populations, in particular isolated populations, could be explained by two hypotheses. The first hypothesis claims that the distribution of genetic diversity within and between populations reflects historical gene flow processes which led to the fragmentation of larger populations [102, 121, 122]. The second hypothesis argues that geographically proximate populations are more efficiently connected by gene flow than populations separated by greater distance. In case of *P*. *patens* we may prefer the first explanation (i.e., signature of past phylogeographic structure) as high values of inbreeding and low levels of migration between populations were reported above, thus we can exclude efficient genetic exchange between the populations studied. Secondly, the split between the Baltic and the Southern group of populations coincide with the border between two biogeographic regions, boreal and steppic biomes in Europe [50], thus implying a phylogeographical pattern. Nonetheless, we could not find a clear phylogeographic picture in our study which may, at least partly, be due to the nature of marker used in this study; microsatellites reflect more recent evolutionary events [93], and if the currently observed decline in genetic diversity is lasting for a longer while now, the lack of a fine phylogeographic pattern is attributable to the lost phylogeographic signal [123, 124]. For a phylogeographic study, we should focus on more conservative regions that still preserve phylogenetic signal.

### Implications for species conservation

Habitat fragmentation and the decline of *P*. *patens* localities led to a dramatic decrease in the size of *Pulsatilla patens* populations in east Central Europe. The results of our study have confirmed parallel decreasing levels of genetic diversity in the analyzed populations. Low levels of genetic diversity and high levels of inbreeding within populations occupying the western part of the species geographic range could have important implications for the conservation of *P*. *patens*. The approach for selecting priority populations for conservation presented here based on neutral molecular markers which are useful for discerning gen flow and evidence of historic events. Populations with high diversity of neutral markers, and high allelic diversity can be considered suitable candidates for high adaptive variation as well [14] and this population are often pointed to conservation [13, 14, 15].

Our genetic analysis confirms that the highest allelic richness and highest number of locally common alleles are founded in the highest population of *P*. *patens*, occupying area greater than 100m^2^. In the Baltic group of populations, which represented cluster 1 and 2, this populations are: Kopna Góra (P4), Kopytkowo (P8), Zabiele (P15)- cluster 1 and Kolimagi (P14)- cluster 2. Taking into account the biological structure of the population it must be emphasized that in this populations the number of flowering and fruit-producing individuals were the higher. In this populations we also observed the highest percentage of seedlings. Seedling recruitment could be correlated with habitat type, because the above populations were reported from xerothermic grasslands. The remaining Baltic populations were found in pine forests, in sun-exposed locations, generally along forest paths. Therefore, conservation actions have to focus on these large populations of the species in Poland.

As for the Southern group of populations, presented the third genetic group most populations seems to be equally large in terms of actual number of individuals. The highest genetic diversity, taking into account allelic richness and highest locally common alleles was detected in populations P-28 (Frumoasa). Unfortunately, we did not collect data on reproductive success in that part of the area, but we observed the presence of viable seeds of the plants in several Transylvanian populations. Nevertheless, the generally low levels of allelic richness and heterozygosity clearly warrants for declining population genetic characteristics of the populations studied, just in line with the reported decrease of the plant in the region [101]. Especially worrying is the fact that the E Ukrainian (P22) and W Russian (P21) populations studied by us, albeit they are laying more to the east and thus closer to the core area of the species, shows similar level of genetic poverty as all other parts of the area studied. This might indicate a general decline of the species, although more populations have to be studied from the eastern European area of *P*. *patens* to get a clearer picture on this phenomenon. Interestingly, slightly lower levels of inbreeding were observed in the Southern group, with actually the lowest value of FIS detected in an ex situ conservation garden population (P25–Bátorliget). Unfortunately, we cannot exclude the possibility that this is an artifact, as the manager of this garden cannot fully exclude the possibility of incidentally including seeds from Transylvanian populations (B. Lesku *ex verb*.).

Despite the slight differences in the characteristic population genetic traits of *P*. *patens* populations studied, the low levels of genetic diversity and high values of the inbreeding coefficient throughout the studied range will compromise the species' ability to adapt to environmental change. To combat with the extinction of this highly endangered species, our data suggest that conservation efforts should focus on the largest populations of *P*. *patens*, representing the three genetic groups identified in our structural analysis. As practical guidance, we can suggest the usage of honeybees to try to facilitate outcrossing during the spring period at these selected populations.

The species, amongst other factors, may suffers from the lack effective pollinators as indicated by high levels of inbreeding. Recently, Biró et al. [125] reported the significant increase of reproductive success of *Himantoglossum adriaticum*, an endangered orchid species of EU conservation importance, when the plants were situated near a honeybee apiary. Probably, the way how conservation can improve the chance of outcrossing in existing *P*. *patens* population, and thus halt the inbreeding depression, is placing bee families nearby them in the spring period.

## Supporting Information

S1 Table SSR data sets used in the analyzes. (XLSX)

S2 Table Diversity information parameters at 6 SSR loci. (DOCX)

S3 Table Null allele frequencies estimated with MICRO-CHECKER (Van Oosterhout et al. 2004) for six microsatellite loci in *P*. *patens*. (DOCX)

S4 Table Matrix of pairwise estimates of FST with ENA correction below the diagonal and FST values without correction (p<0.05) above the diagonal among studied populations (population acronyms as in Table 1). (DOCX)
