# Supplementary material for: Genetic Diversity and Population Structure of the Rare and Endangered Plant Species Pulsatilla patens (L.) Mill in East Central Europe
Source: PLoS One. 2016 Mar 22;11(3):e0151730. doi: 10.1371/journal.pone.0151730 (PMC4803199; doi:10.1371/journal.pone.0151730)
Supplement: S2 Table — (DOCX) [file pone.0151730.s002.docx]

Tab. 2. Diversity information parameters at 6 SSR loci.

|  | N | Na | Ne | Ho | He | uHe | F |
| --- | --- | --- | --- | --- | --- | --- | --- |
| Pul01 | 584 | 15 | 6.405 | 0,021 | 0.844 | 0,845 | 0.923 |
| Pul02 | 586 | 8 | 3,645 | 0,079 | 0,726 | 0,726 | 0,919 |
| Pul03 | 579 | 10 | 6,407 | 0,000 | 0,844 | 0,845 | 1,000 |
| Pul04 | 577 | 8 | 5,660 | 0.092 | 0,823 | 0,824 | 0.943 |
| Pul05 | 573 | 15 | 8,774 | 0,366 | 0,886 | 0,887 | 0,586 |
| Pul06 | 584 | 10 | 6,143 | 0.095 | 0,837 | 0,838 | 0.976 |
