# Supplementary material for: Genetic Diversity and Population Structure of the Rare and Endangered Plant Species Pulsatilla patens (L.) Mill in East Central Europe
Source: PLoS One. 2016 Mar 22;11(3):e0151730. doi: 10.1371/journal.pone.0151730 (PMC4803199; doi:10.1371/journal.pone.0151730)
Supplement: S3 Table — (DOCX) [file pone.0151730.s003.docx]

Supplementary Tab. 3 Null allele frequencies estimated with MICRO-CHECKER (Van Oosterhout et al. 2004) for six microsatellite loci in P. patens

| Locus | Estimated Frequency  of Null Allele |
| --- | --- |
| *Pul*01 | 0,3598 |
| *Pul*02 | 0,3260 |
| *Pul*04 | 0,1671 |
| *Pul*06 | 0,2630 |
| *Pul*10 | 0,3251 |
| *Pul*11 | 0,2791 |
