# Supplementary material for: Genetic Diversity and Population Structure of the Rare and Endangered Plant Species Pulsatilla patens (L.) Mill in East Central Europe
Source: PLoS One. 2016 Mar 22;11(3):e0151730. doi: 10.1371/journal.pone.0151730 (PMC4803199; doi:10.1371/journal.pone.0151730)
Supplement: S4 Table — (DOCX) [file pone.0151730.s004.docx]

| Pop | P1 | P2 | P3 | P4 | P5 | P6 | P7 | P8 | P9 | P10 | P11 | P12 | P13 | P14 | P15 | P16 | P17 | P18 | P19 | P20 | P21 | P22 | P23 | P24 | P25 | P26 | P27 | P28 | P29 |
| --- | --- | --- | --- | --- | --- | --- | --- | --- | --- | --- | --- | --- | --- | --- | --- | --- | --- | --- | --- | --- | --- | --- | --- | --- | --- | --- | --- | --- | --- |
| P1 |  | 0.164 | 0.098 | 0.105 | 0.136 | 0.117 | 0.495 | 0.172 | 0.141 | 0.311 | 0.272 | 0.073 | 0.250 | 0.092 | 0.139 | 0.157 | 0.288 | 0.199 | 0.468 | 0.178 | 0.265 | 0.219 | 0.255 | 0.153 | 0.243 | 0.200 | 0.188 | 0.130 | 0.216 |
| P2 | 0.224 |  | 0.124 | 0.064 | 0.162 | 0.226 | 0.456 | 0.045 | 0.174 | 0.124 | 0.235 | 0.104 | 0.230 | 0.125 | 0.006 | 0.159 | 0.257 | 0.181 | 0.262 | 0.159 | 0.176 | 0.189 | 0.197 | 0.150 | 0.141 | 0.156 | 0.164 | 0.183 | 0.181 |
| P3 | 0.115 | 0.194 |  | 0.063 | 0.062 | 0.115 | 0.373 | 0.124 | 0.125 | 0.219 | 0.099 | 0.085 | 0.120 | 0.046 | 0.097 | 0.129 | 0.168 | 0.085 | 0.320 | 0.072 | 0.177 | 0.148 | 0.184 | 0.110 | 0.171 | 0.125 | 0.136 | 0.097 | 0.119 |
| P4 | 0.115 | 0.099 | 0.100 |  | 0.087 | 0.105 | 0.397 | 0.045 | 0.094 | 0.199 | 0.198 | 0.075 | 0.204 | 0.080 | 0.035 | 0.076 | 0.233 | 0.153 | 0.315 | 0.126 | 0.127 | 0.160 | 0.151 | 0.093 | 0.115 | 0.087 | 0.107 | 0.096 | 0.120 |
| P5 | 0.200 | 0.239 | 0.086 | 0.109 |  | 0.054 | 0.324 | 0.164 | 0.169 | 0.204 | 0.274 | 0.107 | 0.267 | 0.121 | 0.133 | 0.160 | 0.295 | 0.227 | 0.255 | 0.198 | 0.254 | 0.225 | 0.275 | 0.187 | 0.203 | 0.182 | 0.207 | 0.166 | 0.201 |
| P6 | 0.145 | 0.244 | 0.089 | 0.058 | 0.019 |  | 0.476 | 0.201 | 0.124 | 0.249 | 0.301 | 0.169 | 0.314 | 0.154 | 0.172 | 0.173 | 0.336 | 0.263 | 0.406 | 0.232 | 0.267 | 0.250 | 0.299 | 0.193 | 0.273 | 0.188 | 0.219 | 0.128 | 0.229 |
| P7 | 0.443 | 0.383 | 0.233 | 0.261 | 0.215 | 0.353 |  | 0.433 | 0.478 | 0.517 | 0.566 | 0.411 | 0.536 | 0.403 | 0.404 | 0.465 | 0.589 | 0.546 | 0.560 | 0.464 | 0.499 | 0.468 | 0.504 | 0.474 | 0.464 | 0.494 | 0.476 | 0.487 | 0.455 |
| P8 | 0.223 | 0.076 | 0.192 | 0.069 | 0.222 | 0.196 | 0.354 |  | 0.142 | 0.218 | 0.208 | 0.110 | 0.214 | 0.113 | 0.035 | 0.129 | 0.238 | 0.169 | 0.312 | 0.144 | 0.125 | 0.169 | 0.168 | 0.153 | 0.135 | 0.137 | 0.161 | 0.182 | 0.166 |
| P9 | 0.211 | 0.265 | 0.194 | 0.145 | 0.257 | 0.157 | 0.442 | 0.207 |  | 0.271 | 0.207 | 0.178 | 0.207 | 0.062 | 0.104 | 0.156 | 0.252 | 0.166 | 0.385 | 0.096 | 0.206 | 0.209 | 0.235 | 0.157 | 0.185 | 0.170 | 0.213 | 0.133 | 0.222 |
| P10 | 0.406 | 0.131 | 0.257 | 0.202 | 0.264 | 0.278 | 0.503 | 0.244 | 0.340 |  | 0.365 | 0.261 | 0.359 | 0.249 | 0.144 | 0.274 | 0.390 | 0.323 | 0.237 | 0.287 | 0.330 | 0.343 | 0.354 | 0.296 | 0.293 | 0.268 | 0.313 | 0.289 | 0.312 |
| P11 | 0.341 | 0.315 | 0.129 | 0.248 | 0.360 | 0.328 | 0.528 | 0.272 | 0.279 | 0.447 |  | 0.247 | 0.051 | 0.125 | 0.200 | 0.251 | 0.098 | 0.004 | 0.462 | 0.069 | 0.233 | 0.241 | 0.264 | 0.192 | 0.254 | 0.228 | 0.261 | 0.226 | 0.214 |
| P12 | 0.097 | 0.159 | 0.139 | 0.106 | 0.157 | 0.182 | 0.311 | 0.158 | 0.275 | 0.321 | 0.335 |  | 0.231 | 0.097 | 0.109 | 0.109 | 0.278 | 0.200 | 0.359 | 0.155 | 0.236 | 0.194 | 0.240 | 0.163 | 0.218 | 0.177 | 0.158 | 0.154 | 0.204 |
| P13 | 0.331 | 0.321 | 0.170 | 0.268 | 0.363 | 0.364 | 0.506 | 0.287 | 0.287 | 0.450 | 0.074 | 0.325 |  | 0.114 | 0.186 | 0.243 | 0.103 | 0.011 | 0.442 | 0.071 | 0.256 | 0.253 | 0.279 | 0.235 | 0.246 | 0.229 | 0.279 | 0.261 | 0.263 |
| P14 | 0.105 | 0.197 | 0.078 | 0.130 | 0.166 | 0.145 | 0.298 | 0.172 | 0.086 | 0.291 | 0.153 | 0.156 | 0.152 |  | 0.084 | 0.133 | 0.173 | 0.089 | 0.347 | 0.038 | 0.184 | 0.168 | 0.209 | 0.129 | 0.170 | 0.165 | 0.184 | 0.125 | 0.175 |
| P15 | 0.180 | 0.011 | 0.152 | 0.059 | 0.188 | 0.171 | 0.316 | 0.063 | 0.153 | 0.140 | 0.264 | 0.163 | 0.251 | 0.131 |  | 0.131 | 0.224 | 0.148 | 0.260 | 0.115 | 0.134 | 0.167 | 0.153 | 0.122 | 0.103 | 0.133 | 0.142 | 0.152 | 0.155 |
| P16 | 0.233 | 0.224 | 0.180 | 0.090 | 0.230 | 0.219 | 0.418 | 0.170 | 0.236 | 0.343 | 0.330 | 0.149 | 0.335 | 0.183 | 0.178 |  | 0.279 | 0.204 | 0.393 | 0.162 | 0.255 | 0.235 | 0.279 | 0.173 | 0.218 | 0.137 | 0.195 | 0.129 | 0.204 |
| P17 | 0.380 | 0.337 | 0.212 | 0.277 | 0.395 | 0.385 | 0.596 | 0.295 | 0.327 | 0.492 | 0.124 | 0.368 | 0.139 | 0.203 | 0.281 | 0.373 |  | 0.083 | 0.480 | 0.120 | 0.299 | 0.282 | 0.308 | 0.281 | 0.264 | 0.253 | 0.306 | 0.306 | 0.308 |
| P18 | 0.278 | 0.268 | 0.130 | 0.211 | 0.328 | 0.299 | 0.518 | 0.243 | 0.245 | 0.422 | 0.024 | 0.299 | 0.019 | 0.131 | 0.214 | 0.302 | 0.105 |  | 0.422 | 0.050 | 0.197 | 0.195 | 0.231 | 0.177 | 0.208 | 0.180 | 0.228 | 0.209 | 0.206 |
| P19 | 0.500 | 0.194 | 0.303 | 0.265 | 0.290 | 0.375 | 0.561 | 0.279 | 0.408 | 0.238 | 0.474 | 0.362 | 0.466 | 0.332 | 0.207 | 0.420 | 0.519 | 0.442 |  | 0.377 | 0.424 | 0.385 | 0.451 | 0.396 | 0.357 | 0.393 | 0.430 | 0.425 | 0.407 |
| P20 | 0.246 | 0.253 | 0.123 | 0.196 | 0.289 | 0.264 | 0.412 | 0.221 | 0.142 | 0.363 | 0.084 | 0.251 | 0.098 | 0.062 | 0.181 | 0.239 | 0.152 | 0.082 | 0.388 |  | 0.201 | 0.179 | 0.214 | 0.164 | 0.180 | 0.174 | 0.212 | 0.175 | 0.201 |
| P21 | 0.354 | 0.267 | 0.232 | 0.163 | 0.353 | 0.303 | 0.492 | 0.179 | 0.278 | 0.418 | 0.289 | 0.328 | 0.328 | 0.231 | 0.199 | 0.343 | 0.378 | 0.263 | 0.461 | 0.265 |  | 0.114 | 0.052 | 0.115 | 0.094 | 0.113 | 0.103 | 0.199 | 0.081 |
| P22 | 0.308 | 0.267 | 0.197 | 0.197 | 0.328 | 0.295 | 0.465 | 0.219 | 0.283 | 0.437 | 0.297 | 0.281 | 0.323 | 0.214 | 0.224 | 0.325 | 0.366 | 0.261 | 0.410 | 0.238 | 0.132 |  | 0.128 | 0.157 | 0.172 | 0.163 | 0.139 | 0.196 | 0.156 |
| P23 | 0.348 | 0.295 | 0.233 | 0.185 | 0.380 | 0.356 | 0.521 | 0.234 | 0.313 | 0.455 | 0.339 | 0.330 | 0.365 | 0.261 | 0.223 | 0.377 | 0.410 | 0.323 | 0.507 | 0.285 | 0.084 | 0.163 |  | 0.097 | 0.067 | 0.138 | 0.110 | 0.204 | 0.090 |
| P24 | 0.228 | 0.233 | 0.158 | 0.118 | 0.272 | 0.228 | 0.461 | 0.220 | 0.229 | 0.381 | 0.269 | 0.249 | 0.329 | 0.181 | 0.183 | 0.254 | 0.383 | 0.273 | 0.439 | 0.245 | 0.166 | 0.222 | 0.128 |  | 0.094 | 0.103 | 0.050 | 0.040 | 0.033 |
| P25 | 0.328 | 0.214 | 0.215 | 0.139 | 0.283 | 0.316 | 0.448 | 0.196 | 0.261 | 0.373 | 0.324 | 0.300 | 0.323 | 0.219 | 0.154 | 0.295 | 0.353 | 0.291 | 0.395 | 0.250 | 0.147 | 0.221 | 0.101 | 0.119 |  | 0.090 | 0.134 | 0.179 | 0.109 |
| P26 | 0.293 | 0.237 | 0.181 | 0.114 | 0.273 | 0.233 | 0.473 | 0.201 | 0.259 | 0.344 | 0.310 | 0.265 | 0.316 | 0.233 | 0.193 | 0.206 | 0.352 | 0.271 | 0.428 | 0.257 | 0.155 | 0.226 | 0.185 | 0.142 | 0.113 |  | 0.080 | 0.086 | 0.087 |
| P27 | 0.264 | 0.246 | 0.183 | 0.128 | 0.302 | 0.260 | 0.467 | 0.225 | 0.293 | 0.397 | 0.340 | 0.236 | 0.368 | 0.240 | 0.198 | 0.269 | 0.404 | 0.321 | 0.476 | 0.293 | 0.146 | 0.201 | 0.138 | 0.060 | 0.116 | 0.102 |  | 0.086 | 0.053 |
| P28 | 0.183 | 0.251 | 0.133 | 0.116 | 0.237 | 0.169 | 0.430 | 0.239 | 0.200 | 0.355 | 0.290 | 0.219 | 0.340 | 0.170 | 0.203 | 0.182 | 0.388 | 0.287 | 0.441 | 0.244 | 0.243 | 0.246 | 0.243 | 0.039 | 0.203 | 0.103 | 0.091 |  | 0.079 |
| P29 | 0.302 | 0.262 | 0.159 | 0.143 | 0.288 | 0.271 | 0.436 | 0.225 | 0.307 | 0.393 | 0.293 | 0.288 | 0.356 | 0.232 | 0.215 | 0.279 | 0.408 | 0.303 | 0.449 | 0.283 | 0.118 | 0.193 | 0.122 | 0.049 | 0.130 | 0.110 | 0.062 | 0.092 |  |

Table 4 Matrix of pairwise estimates of FST with ENA correction below the diagonal and FST values without correction (p<0.05) above the diagonal among studied populations (population acronyms as in Table 1)
